# Current and Future Costs of Family Caregiving for Older Americans With and Without Dementia

**DOI:** 10.1093/geroni/igaf122.601

**Published:** 2025-12-31

**Authors:** Stipica Mudrazija, María Aranda

**Affiliations:** University of Washington, Seattle, Washington, United States; University of Southern California, Los Angeles, California, United States

## Abstract

Family caregivers in the US provide substantial value of unpaid care to older adults, while less recognized are the employment-related costs they endure and the trajectory of these costs. We estimate the replacement cost of unpaid family caregiving to US adults aged 70 and older with and without dementia and opportunity costs of forgone earnings, lost productivity, and loss of federal income tax revenue between 2011 and 2060, using data from the National Study of Caregiving, Panel Study of Income Dynamics, and various other sources such as Census Bureau’s population projections. Currently, the annual replacement cost of unpaid family care is between $96-182 billion, with 44% accounted for by dementia caregiving. By 2060, it will increase to $277-571 billion, and 53% will be for dementia caregiving. The opportunity costs of forgone earnings, productivity loss, and loss of federal income tax payments, however, will grow faster, increasing from current levels of $107 billion, $26 billion, and $7 billion to $380 billion, $102 billion, and $27 billion, respectively, in 2060. These costs will be increasingly borne by minoritized caregivers. For example, Latino caregivers will account for 48% of replacement costs and 39% of opportunity costs of caregiving for older adults with dementia in 2060, compared to their current share of only 27% and 19%, respectively. These results highlight a growing need for robust and culturally tailored support for unpaid family caregivers to support their loved ones while remaining attached to the labor force.

